# Supplementary material for: Brain-optimized extraction of complex sound features that drive continuous auditory perception
Source: PLoS Comput Biol. 2020 Jul 2;16(7):e1007992. doi: 10.1371/journal.pcbi.1007992 (PMC7363106; doi:10.1371/journal.pcbi.1007992)
Supplement: Attachment — Submitted filename: Berezutskaya_SA_Ramsey_resubmission_review.pdf [file pcbi.1007992.s002.pdf]

The authors have made a number of efforts to clarify the methods of the paper. I particularly appreciated the dramatic improvements to Figure 1, which is both much clearer and provides some useful insights as to which aspects of the model improved performance.

I continue to think this paper represents a useful methodological advance, and is worth of being published as a methods paper. However, the scientific claims are not supported by the data. The key claims from the abstract are:

"Several features encoded speech-specific acoustic properties with some features exhibiting shorter latency profiles (associated with responses in posterior perisylvian cortex) and others exhibiting longer latency profiles (associated with responses in anterior perisylvian cortex). Our results support and extend the current view on speech perception by demonstrating the presence of temporal hierarchies in perisylvian cortex and involvement of cortical sites outside of this region during audiovisual speech perception."

What is the evidence for speech-specificity? The fact that a few electrodes on average respond preferentially to speech over music (Fig 5b)? What representation wouldn't show this? I suppose there is the fact that the model better predicts speech in Movie II, but it's unclear what to make of this, especially given that the same is not true in Movie I.

With regards to the posterior-to-anterior hierarchy, the only quantitative data plotted is in the figure below, which is from the response and not even included in the paper. The figure shows a trend line for five hand-picked features. The trend line looks like it accounts for a tiny fraction of the variance, and seems to be driven by maybe the first or second feature.

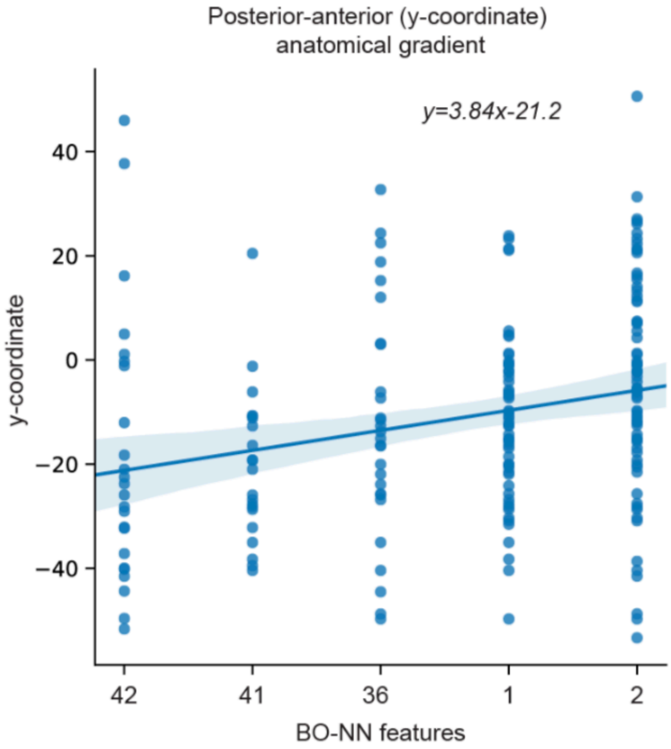

Finally, the "audiovisual speech" effect could be driven entirely by correlations in the stimulus set between visual and auditory features (i.e. the neural responses themselves might be entirely tuned to one modality alone). The fact that the effect cannot be explained by

differences in luminance is uncompelling as the visual cortex responds to an enormous range of visual features beyond luminance.

The paper would be much stronger in my opinion if it was purely methodological. The scientific claims are not supported by the data, and water down an otherwise useful methodological advance.

--

I have one other substantial methodological comment. When describing the STMF baseline, the authors note that they considered a wide range of parameters for frequency, temporal and spectral modulation. But the authors do not say anything about including temporally delayed copies of the response, which is obviously important when trying to predict a time-varying signal. Can the authors comment on this, and if necessary run a version of the analysis that includes temporal delays?

I have a similar worry with the RSA analysis (which the authors have done a nice job of clarifying). Since the model and brain data are being compared timepoint-by-timepoint, trivial differences in delays could explain the better performance of the DNN model. Or perhaps the STMF just doesn't have the right proportion of each type of spectrotemporal modulation filter? The authors could try and address this by performing RSA after a linear transformation to align the model with the data. But I honestly don't see any benefit of the RSA analysis over simply predicting the response.

Minor comments:

1. In Figure 7b, the kernel density plots don't seem to reflect the correlation plots below. For example, the spectral modulation correlations appear broadly distributed in the lower plot, but peaked in the kernel density plot.

2. Why go with the dual stream model as the main model, given that it is more complex, and doesn't seem to predict responses better? The authors say "The reason for that was that we aimed to maximize the generalization capacity of the ANN and considered that both types of input may carry information that could aid generalization." But they do not provide any evidence that the more complex model aids in generalization.

3. The authors' measure of speech and music selectivity is still suboptimal, since it doesn't obviously measure whether the responses are consistently higher or larger for speech or music. I suggested using d-prime, but the authors responded saying: "We reasoned that the d-prime score is more suitable for discrete responses." What I meant was to simply measure the difference in the means divided by standard deviation of the response within each category, which is a very standard way of measuring the amount of overlap between two continuous distributions.

4. There are small grammatical and text errors throughout. I have not noted them all because this would have been time consuming, but hopefully the paper could be carefully proofread before publication.
