# Supplementary material for: Brain-optimized extraction of complex sound features that drive continuous auditory perception
Source: PLoS Comput Biol. 2020 Jul 2;16(7):e1007992. doi: 10.1371/journal.pcbi.1007992 (PMC7363106; doi:10.1371/journal.pcbi.1007992)
Supplement: Attachment — Submitted filename: response_to_reviewers-nfr.docx [file pcbi.1007992.s001.docx]

*************************************************************************************************************************************************

**The text in red is part of the revised manuscript (reference to the corresponding page mentioned). The text in orange is comments to the reviewers that are not part of the manuscript.**

**Please note that because we added an extra figure to the revised manuscript (Figure 2) the figure numbers the reviewers refer to do not always correspond. For example, old Figure 6 in the reviewer’s comment is a new Figure 7, old Figure 2 is a new Figure 3. Old Figure 1 remains Figure 1.**

*************************************************************************************************************************************************

**Reviewer #1**

In “Brain-optimized extraction of complex sound features that drive continuous auditory perception”, Berezutskaya et al. use a “brain optimized neural network” (BO-NN) to discover features driving neural activity recorded from ECoG during a movie watching task, and then use those features to predict neural data recorded from different participants on a separate natural listening task. They train a recurrent convolutional neural network to extract feature representations while 6 ECoG patients watch a Dutch movie. The model incorporates information from the raw audio waveform as well as 2D audio spectrogram features, and is optimized to predict responses to a full length feature movie with both auditory and visual information. The major novelty of this manuscript is in the ability to predict data from a completely separate cohort of participants listening to different movies. In particular, the authors show that their model captures responses to speech, but that responses to musical portions of the second stimulus set (Movie 2) were not as readily predictable. It is also very interesting that they are able to predict the responses in some higher order (presumably non-spectrotemporal) regions such as IFG, MTG, and postcentral gyrus. While I believe the results of this study will be interesting to readers of PLoS Computational Biology and I am enthusiastic about the use of a data-driven set of model features on an entirely new dataset, my main suggestions relate to interpretation of the neural network model features. There are a few details that I believe would aid in interpreting their results, especially with regard to comparing performance on speech vs. music tasks.

**Major comments:**
1. The differences in performance in music and speech are interesting, but I wonder whether these are driven by inherent differences in the acoustics of the training and test data. For example, in Figure 1, where only data from Movie 1 is used, the authors can predict responses to all classes of sounds, including music. However, once Movie 2 stimuli (alternating speech and music) are used, the performance of the model on musical stimuli decreases significantly. This leads me to a few questions:

a. Is there equal occurrence of speech and music in Movie 1 training set?

There was overall more speech (39% of total movie duration) than music (16% of total movie duration) in the soundtrack of Movie 1 (**Figure 1a**).

b. Are differences in speech and music predictions driven by similarity of speech stimuli from movie 1 and 2 and differences in acoustics for music in movie 1 and 2? An acoustic analysis (similarity of modulation spectra for each sound class in Movie 1 vs. 2) would be helpful here.

The majority of music fragments in Movie 1 were instrumental (96%), while only a small portion contained lyrics (4%, Figure 1a). The music fragments of Movie 2 were all instrumental. There was in total more music in Movie 1: ~15% of 78 minutes (total duration) constitutes ~12 minutes of music compare to Movie 2: only 3.5 minutes. The number of individual music pieces in Movie 1 was much bigger as there were 120 separate music fragments (separated from each other by other sound types) in Movie 1 compared to only seven separate music blocks in Movie 2. As Movie 1 had more individual music fragments, the overall diversity of music material was also bigger there compared to seven 30-second music blocks in Movie 2.

In order to test the similarity of the acoustic features of Movie 1 and of Movie 2 we performed the following analysis: we selected a sound sample (one sound data point at 50 Hz) from Movie 2 and computed the cosine similarity of its spectrotemporal representation (STMFs) with all sounds of the same type (music or speech) in Movie 1, noting down the value of the maximal cosine similarity. The process was repeated 1,000 times for music sound samples (only compared with music in Movie 1) and 1,000 times for speech sound samples (only compared with speech in Movie 1). We found that on average the similarity of spectrotemporal features between Movie 1 and Movie 2 was similar across speech and music: $\bar{s}_{speech}=91\pm.02$ and $\bar{s}_{music}=.89\pm.03$. As a result of this analysis, we consider it unlikely that the acoustic difference between music in Movie 1 and Movie 2 led to the difference in generalization of the BO-NN model to speech and music data of Movie 2. Yet, we cannot fully exclude differences in sound features in music across movies.

c. Could the authors comment on the music stimuli in Movie 1 vs. Movie 2 in terms of whether they use similar instruments, contain vocals or not, are similar genres, etc? Because music is a very broad category, it could be that results will not generalize to music unless music that was acoustically similar shows up in the training set (Movie 1).

We think that there is another explanation to why we observed the difference in predicting music of Movie 1 compared to music of Movie 2. We believe that lack of generalization may be explained by differences in HFB cortical responses to music in Movie 2 due to attention shifts and other top-down processes. Specifically, soundtrack of Movie 2 had a block design and the subjects could essentially predict the switches between music and speech fragments. Speech fragments contained dialogues relevant for the development of the story whereas music pieces did not. This contrast and the predictable block structure of the soundtrack could lead to an attentional shift towards speech blocks. In fact we observed that the HFB responses during speech blocks were on average higher compared to music blocks: 49% of superior temporal gyrus electrodes and 17% of inferior frontal gyrus electrodes had positive mean $r^{2}$ values of $.77$ (mean$t = 59.83$) and $.7$(mean $t = 32.03$), respectively (in this analysis $r^{2}$ was calculated between each electrode’s HFB and music/speech binary vector with ones for speech blocks and zeros for music blocks). This was in spite of the fact that the sound intensity of music blocks was on average higher compared to speech blocks: signed $r^{2}= -.31, t = -4.54, p = 5.4 \times{10}^{-4}$. There is literature that shows that attentional shifts and predictive mechanisms can affect the way neuronal populations encode perceptual information (Alain & Woods, 1997; Sabri et al., 2008; Shamma et al., 2011; Talsma et al., 2010). It requires further, more detailed investigation to understand how ANNs could tackle such issues and learn to capture attention related effects in the cortical responses.

We mention these considerations on p. 7 of the Results.

2. Why was RSA used to compare the neural network models to the STG data? It would be more straightforward to compare the correlations directly for the random-NN, BO-CNN, and STMF models for the STG electrodes in the same way that this was done for non-STG electrodes. The correlations could be compared separately for music and speech and would be more easily interpreted than the RSA analysis.

**As STG is the main region of higher-level associative cortex involved in sound processing we aimed to determine whether an ANN optimized to predict brain responses to sound can learn representations similar to STG. RSA is often used for comparing representational geometries across modalities and models, and therefore we applied it in our case as well. We also used it for comparing representations in other cortical regions, including IFG, Precentral and Postcentral regions and middle temporal gyrus. We did not observe a better similarity between ANN and cortical representations in these regions compared to STMF and random-NN there. It is possible that the ANN was biased to fit STG responses in particular because a large number of ECoG channels in training dataset came from STG (47 electrodes) and both the amplitude and variance of response to sound were on average higher in STG compared to other regions:**

**We now report direct comparisons of correlations between predicted and observed STG responses in the text (p 6.): *“Compared to both STMFs and Rand-NN, top-layer BO-NN reached higher prediction accuracy and fitted more electrodes not only in STG (two-sided Wilcoxon signed-rank test: Z_(BONN-STMF)=4.34,p<.001, +8 sign. els., Z_(BONN-RANDNN)=8.94,p<.001, +49 sign. els.), but also in the higher-level speech processing areas …”***

**In addition, we added a plot with prediction accuracy values per STG electrode for the random-NN, BO-CNN, and STMF model in **Figure 4a**.**

3. Regarding the key features analyses, I think it is a great idea to try and correlate these with known quantities in an effort to better understand what the neural network has learned. However, I found this part of the manuscript to be the weakest part, since no specific suggestions or interpretations were made for each of the key features listed. I thus have a few specific questions:

a. Are key features 1, 2, and 36 (the speech specific features) actually tuned to any particular speech features? (e.g. certain spectrotemporal modulations, phonetic content, etc)

In speech the features #1, #2 and #36 are all strongly correlated with some of the STMFs: $\rho_{max}=.69$, $\rho_{max}=.69$ and $\rho_{max}=.69$, respectively. The STMF correlation profiles for features #1 and #2 are shown in Figure 8. The STMF correlation profile for feature #36 is shown below:

During music fragments features #1 and #2 are similarly correlated with STMFs: $\rho_{max}=.7$, $\rho_{max}=.66$, feature #36 however is correlated substantially less: $\rho_{max}=.42$ (see below):

This can be explained by the fact that key feature #36 appears to be more tuned to higher temporal modulations (TMs, > 4 Hz, see plot for speech), whereas music fragments contained slower temporal modulations (see below) and were therefore better captured by key features correlated with slow temporal STMFs:

b. Key feature 35 is a strange one. Is it ever off, or is it constantly on in a sustained fashion (as described in the text)? It would make more sense if this was a “sound on” vs. “off” feature, but more description is needed.

We looked at feature #35 more closely. It appears to follow an interesting shape with moments of sustained high response to long fragments of the soundtrack (each vertical black line represents the border between the sound blocks):

We adjusted the description of the feature’s time course in the text (p. 10):

*“One feature exhibited sustained and slowly changing response to sound, often climbing throughout a music block and dropping throughout a speech block (key feature #35).”*

c. Key feature 42’s cortical weight maps seem quite weak for music – is this real, or is this for a particular type of music (such as music with vocals?)

All music in Movie II was instrumental. Indeed, Figure 5b shows that key feature #42 exhibits somewhat weaker response to music compared to speech.

d. Key features 2 and 15 are described as being related to differences in speech vs. music – in particular, their activation time courses were less anticorrelated during music compared to speech. Could the authors provide some additional discussion related to this point? What exactly might it be about the speech vs. music stimuli that drives this?

Both features were associated with cortical activity in perisylvian cortex and LOTC. Feature #15 was correlated with activity in LOTC and anticorrelated with perisylvian cortex and feature #2 showed the reverse profile: it was correlated with activity in perisylvian cortex and anticorrelated with LOTC. We believe that pattern of relations between the two cortical regions have to do with the interaction of the auditory and visual domains in the movie. In fact, in a different project we analyze the same dataset with respect to the visual processing and report response within LOTC and parietal regions to landscape frames and frames containing movement. It is possible that speech fragments are more associated with presence of faces in relatively static movie frames compared to absence of speech, which in turn is more likely during absence of face close-ups, but presence of human action, general movement or on the contrary landscape frames that are free of people. Full investigation of the degree and nature of these interactions is beyond the scope of the current paper.

We added these observations to the Discussion (p. 16).

4. I’m not sure I understand Figure 6b. How are the density plots related to the black lines? Are they simply fits to the data shown by the black lines? If so, is it the case that the BO-NN features only represent relatively high spectral modulations and no low spectral modulations? Are these low spectral modulations also not informative of the neural data? I’m surprised, as I would think that these, coupled with high temporal modulations, would predict some regions of posterior STG.

We updated the plot in **Figure 7b** and separated the kernel density estimation plots from the correlation plots to make the graphs more visible. Per BO-NN feature we find an STMF with the most similar time course. Each STMF is a combination of values along three dimensions: spectral modulations (SMs), temporal modulations (TMs) and frequency. Therefore, per BO-NN feature we plot the correlation value (a dotted line) for the value of each STMF dimension separately. The procedure is done for speech and music separately.

Above the correlation plots we also show the Gaussian kernel density plots and the rug plots for the STMF values that individual key BO-NN features are ‘selectively tuned to’. Effectively, instead of associating each BO-NN feature with a combination along all three STMF dimensions we estimate which of the three dimensions the given BO-NN feature is most selective to (see the legend and the description in Methods, p.29). It turns out that there is less selective tuning to the spectral modulations in speech than the temporal modulations, possibly because the variation along the dimension of the temporal modulations is likely linked to the language units of varying lengths (such as phonemes, syllables, words and phrases).

5. Similarly, I do not understand what Figure 7a is showing. Is this the time of the peak lag in the autocorrelation? If so, this doesn’t seem to completely match with the analysis in Figure 7b, which also shows the timescale of correlations with Praat features. How are the key features sorted? Please clarify.

In **Figure 8a** we showed the autocorrelation profiles per feature by representing the ‘knee of the autocorrelation’ defined as the time point at which the autocorrelation drops below .5. As pointed out by another reviewer, such binary representation is uncommon, so we added the continuous traces of autocorrelation to the same plot. The order of the features is the same as on the neighboring plot (Prediction of PRAAT features). This was done so that it was easier to observe the autocorrelation value and the latency profile of each feature. Some features (#41, #42, #36, #1, #15 and #2) are highlighted in both plots.

In addition we reported that the correlation between the autocorrelation value and the peak lag for predicting speech properties (PRAAT features) over features does not reach significance ($\rho=.2, p=.17$), confirming that both temporal characteristics of BO-NN features are decoupled.

6. With regard to Figure 7b, this figure would be much more helpful if it was clear which specific features (intensity, pitch, speech ON, or multiple) were contributing to each feature. Are they separable?

We reported some observations of tuning to specific Praat features based on the regression weights (Results, p.12):

*“Some features did stand out for speech. For example, key feature #41 showed a peak response to speech intensity -50 – 0 ms prior to the onset. Key feature #1 was most tuned to speechON at a lag of 200 – 350 ms, and key feature #2 – at a lag of 250 – 300 ms. Some features were associated with negative weights for speech being on and sound intensity in general, and thus were “deactivated” by speech (key features #15 and #26).”*

At the same time, because all these features (intensity, pitch, speechON) are very correlated in the continuous stream of speech it is hard to make conclusive statements regarding their separability in the BO-NN features or the neural responses.

7. The authors should comment on how their mapping is or is not consistent with recent findings from other groups (e.g. Norman-Haignere et al. Neuron 2016, which showed posterior and anterior regions selective for non-vocal music that were separate from speech selective areas of the STG). This paper also showed some differential responses for music with and without singing, which could inform the question posed in #1 above.

We find this point more difficult to address in our setup with a continuous naturalistic audio stimulus with an unbalanced distribution over different types of sounds (Movie I). We report Figure 2b because we think the readers will be curious about how well responses to different types of sound are modeled. At the same time, performing formal analyses did not show sound selectivity across electrodes. It appeared that more or less the same electrodes in STG were fitted well by the BO-NN model regardless of the type of sound.

The soundtrack of Movie II was manipulated by inserting interleaved blocks of speech and music. Performing a sound selectivity analysis there showed preference to speech fragments over music in many STG and IFG electrodes. However, as discussed previously, we think that the cortical responses to music in Movie II were modulated by the attentional shifts and therefore we are not sure if any claims regarding sound selectivity in any electrode could be made.

Altogether, we think that neither movie stimulus used here is suited for this kind of analysis. Instead, we would propose to address sound selectivity on another, more experimentally controlled dataset. In that case, the sound data from separate trials (of presenting different sounds) can be passed through the BO-NN and the neural responses can be fitted using the resulting BO-NN representation in the same way we did here for the generalization dataset of Movie II. It could be an interesting follow-up study.

**Minor comments:**
1. The statement in the abstract “The extracted bottom- up features captured acoustic properties that were specific to the type of sound and were associated with various response latency profiles and distinct cortical distributions” is quite vague. The authors should expand this to provide specific examples that they found from their results, or remove the sentence entirely.

We added the following sentence:

*“Specifically, several features encoded speech-specific acoustic properties with some features exhibiting shorter latency profiles (associated with responses in posterior perisylvian cortex) and others exhibiting longer latency profiles (associated with responses in anterior perisylvian cortex).”*

2. In Figure 1, it could be helpful to connect the dots for each electrode in Figure 1b, since although the overall performance on speech vs. music, noise, and ambient sounds did not differ, it could be that those differences are observed in individual electrodes, with some tuned for different classes of sound.

**We updated the figure by connecting the dots for each electrode. We also added a figure (**Figure 2c**) for the best fitted electrode showing the distribution of prediction accuracy over the fragments of each sound type (30-second fragments were selected per sound type at random and prediction accuracy was calculated for each of them).**

**As mentioned in the text, we did not observed sound selectivity across the electrodes. At the same time, the continuous nature of the movie complicates the analysis.**

3. The “transverse gyrus” is not standard nomenclature – it would be clearer to call this either the “transverse temporal gyrus” or “Heschl’s gyrus” or perhaps “transverse gyrus of Heschl”.

**We replaced all mentions of ‘transverse gyrus’ with ‘Heschl’s gyrus’.**

4. The authors note that the movie clips in Movie 2 were dubbed, so presumably the visual does not match the auditory stimulus. Perhaps they could comment on whether this influences any of the interpretations of their results.

It is possible that our use of a dubbed soundtrack in Movie II affected the ECoG responses to speech for example, through the McGurk effect, which represents interaction between visual and auditory speech modalities (McGurk & MacDonald, 1976). However, we believe that it is unlikely to have influenced the specific tuning of different cortical regions to the auditory features and the latency profiles of the BO-NN features because the reported results are in line with the previous work on auditory perception (Hullett et al., 2016) and the audiovisual perception (Cate et al., 2012).

5. The statement “code will be available upon reasonable author request” – I have seen this statement before, but authors were asked to change it to “upon request from the corresponding author”, as the word “reasonable” implies some value judgment on part of the authors, and the code should be made readily available. Data, on the other hand, could be made available through a University agreement so as to adequately protect patient related health information.

**We rephrased the statement and it now reads: ‘The data supporting the current study have not been deposited in a public repository due to the restrictions on public sharing of the patients’ data but are available from the corresponding author on request. The code containing the BO-NN architecture and the details of training is available at** [**https://github.com/Immiora/bonn_auditory_perception_ecog**](https://github.com/Immiora/bonn_auditory_perception_ecog)**.’**

**In addition, we are currently writing a dataset paper, along with which we will released the anonymized neural data used in the present study in the open database (**[**www.openneuro.org**](http://www.openneuro.org)**).**

*************************************************************************************************************************************************

**The text in red is part of the revised manuscript (reference to the corresponding page mentioned). The text in orange is comments to the reviewers that are not part of the manuscript.**

**Please note that because we added an extra figure to the revised manuscript (Figure 2) the figure numbers the reviewers refer to do not always correspond. For example, old Figure 6 in the reviewer’s comment is a new Figure 7, old Figure 2 is a new Figure 3. Old Figure 1 remains Figure 1.**

*************************************************************************************************************************************************

**Reviewer #2**

This study uses a neural network trained on ECoG patients watching a movie without strong theoretical assumptions to predict neural response from auditory stimulus. The study shows better prediction power compared to a specific model (spectrotemporal modulation) and analyzes the properties of the nodes in the final layer to shed light on the cortical properties of speech processing. Overall, it is an interesting study with some stimulating findings. For example, the set of properties that is represented by the network and sensitivity to spectrotemporal modulation, pitch, intensity, etc. is intriguing. On the other hand, given that the main conclusions of the paper rest on the properties of the representation in node activation of the network, more controls are needed to make the claims of the study more compelling.

1. It is not entirely clear why a multi-layer neural network is needed to show the claims of the study. The main point of the paper is the superiority of data-driven models over theory-driven models. For this argument, the data-driven model can be any regression framework, for example, STRF (linear), MID (Sharpee et. al.), or even a single-layer neural network. Given that all the analysis is done on the last layer of the network, why exactly the authors claim that multilayer hierarchical feature extraction is needed, and how the claims are dependent on this particular choice is unclear.

We agree that we should not claim that the ANN structure used in this paper is necessary for achieving good prediction accuracy. Given the limited amount of our brain data, we decided against a thorough parameter optimization procedure and instead explored a limited number of alternative architectures. We now made sure that in the manuscript we do not claim that our model architecture is optimal considering all possible architectures. However, based on the limited comparisons that we did perform, the results obtained with the BO-NN model, were the best (Figure 1a). Specifically, we were able to show that a more shallow ANN with a matched number of parameters performed significantly worse compared to the deeper BO-NN model.

To expand on how we made the choices about the architecture of the model we added a separate section to Results (p. 4) and Discussion (p. 14).

2. This particular network architecture (conv + RNN) was chosen because it had the best prediction accuracy. What is the effect of this model choice on the findings? E.g. do they not see the same acoustic features in the last layer of the random network (sensitivity to pitch, music, etc.) which can happen due to the random templates of the stimulus?

We ran two additional analyses on the top layer features of the Rand-NN: a regression to predict STMF and a regression to predict Praat features (intensity, pitch, formants and so on). In addition, we plotted the top Rand-NN features by sorting the difference in their average activation amplitude between music and speech blocks (analogous to the plot in Figure 5b on top BO-NN features).

These results (see figure below) demonstrate that a deep Rand-NN, repeating the RCNN structure of the BO-NN model captures various acoustic properties of the stimulus. After all, it performs the same computations on the audio as the BO-NN but with different weights (see a). At the same time, Rand-NN fails to extract features tuned to specific types of sound (music and speech). The pattern of feature activations seems a lot more random (see b below) compared to the BO-NN model. Finally, Rand-NN does not capture the specificity of the neural response to the audio features, such as the latency of the neural response (see c below). These are just some limited analyses and they might not fully uncover the differences between the random templates of the stimuli (Rand-NN) and the model optimized on the brain data (BO-NN), but they show a much larger capacity of the BO-NN model to capture the brain-related information compared to the random templates.

3. While it is interesting that the STM features are preserved in the neural network, it is perhaps more interesting to show what is added by the network that wasn’t present in theory-driven features. In other words, what is it that the network learns from the data that wasn’t already predictable from STM features? This information is important for the claim of the paper (advantage of data driven over theory)

**It is very hard to pinpoint what is captured in the BO-NN features that helps the model ourperform STMFs. Of the things that we observed, the BO-NN model is capable to learn the temporal profile of the cortical response to the auditory information. It is likely that the combination of convolutional and recurrent processes within 6 seconds of audio data allow the ANN to do so.**

**Apart from the temporal profile of the neural response, using the representational similarity analysis (RSA) on we showed that BO-NN features (in *top*, or recurrent, layer) provide an audio representation that is closer to speech encoding in STG compared to STMFs: BO-NN similarity to STG is was on average .37 compared to only .24 of STMFs. It is therefore likely that BO-NN learns more about the nature of the neural dynamics during auditory processing than is represented in STMFs.**

4. The majority of the claims are based on single node response properties in the last layer of the neural network. While it is shown here that the population of nodes perform well in predicting the neural responses (linear projection of the last layer to the neural response), it is not clear whether the exact node properties are essential to the prediction, or whether they are preserved across different network implementations. In other word, different initialization of weights, different regularization of the network, different architecture (Conv, RNN, …), etc., can all result in the same input-output function and the information in the last layer (hence same prediction power from its linear projection), but the exact information that is represented in each node may not be the same and can change with changing network properties. Hence, the claim of the paper about the node properties is not very compelling unless the authors can show that these exact response selectivity by nodes is “necessary” for successful prediction of the neural data from the last layer, or at least consistently appear across models.

In response to the comment, we first add **Figure 1b** showing significant difference in performance obtained by models using CNN, RNN or RCNN architecture. In the comparison we do not just use one model of each type, but essentially 10 models per type as 10 models are trained each time to cross-validate performance accuracy. Based on the results, we tend to think that RCNN structure provides superior prediction accuracy compared to simple CNN or RNN architectures.

We also observed that the rand-NN fitted the brain responses significantly worse compared to the BO-NN model (based on RSA and scatter plots of accuracy). In addition, plotting the average accuracy of the rand-NN predictions (all 300 features) as a baseline to the clustering showed that using a very small number of BO-NN features (~10) results in the same accuracy as rand-NN:

This means that remaining features encode information relevant for a better fit of the neural data and possibly reflect the characteristics of the neural dynamics and neural response to speech.

We believe that altogether these results suggest that both a better architecture (convolutional and recurrent nodes) and training on the brain data contribute to the model performance and its ability to capture audio representations that match the neural responses.

*************************************************************************************************************************************************

**The text in red is part of the revised manuscript (reference to the corresponding page mentioned). The text in orange is comments to the reviewers that are not part of the manuscript.**

**Please note that because we added an extra figure to the revised manuscript (Figure 2) the figure numbers the reviewers refer to do not always correspond. For example, old Figure 6 in the reviewer’s comment is a new Figure 7, old Figure 2 is a new Figure 3. Old Figure 1 remains Figure 1.**

*************************************************************************************************************************************************

**Reviewer #3**

This manuscript is an interesting attempt to directly train a non-trivial DNN model to predict ECoG responses using about an hour of data from 6 patients collected during the viewing of a natural movie. The authors show the trained model outperforms a baseline spectrotemporal model (and an untrained model) in predicting responses to a shorter movie recorded in a different set of subjects (after linear re-mapping). The authors then take advantage of the fact that many more subjects were tested in the second movie in order to try and understand the organization of feature tuning in auditory cortex and higher-order regions using the model. The primary approach used is to cluster the learned features from the top-level of the trained model and to then investigate the selectivity of these features for different acoustic features or categories, and the extent to which different electrodes weight on these feature clusters.

Overall, I think there is promise in developing better training methods to directly predict neural responses and interpret the resulting features. At the moment, I am not sure there is a clear scientific conclusion that can be drawn from this work. But I think the approach is methodologically new enough that any progress along these lines is a useful contribution, with clearer scientific conclusions perhaps emerging down the road.

Focusing on the results highlighted in the abstract, the authors state “The extracted bottom-up features captured acoustic properties that were specific to the type of sound and were associated with various response latency profiles and distinct cortical distributions.” How could this be otherwise unless cortical selectivity was completely uniform?

We perceive this result as validation of the chosen methodology. Seeing that the chosen method confirms the topographical organization of the cortex may not be surprising but it is important in the context of a novel method.

They also state: “Our results also support and extend the current view on speech perception by demonstrating the presence of temporal hierarchies in perisylvian cortex and involvement of cortical sites outside of this region during audiovisual speech perception.” Figure 7, which is the data behind this claim, is interesting, and provides evidence that features with more delayed responses also have slower responses with stronger autocorrelations and coarser modulation tuning. I am not sure I find this result particularly surprising, but it’s a useful demonstration of how one can use the method. The supposed anatomical progression in Figue 7C is not particularly compelling in my opinion and is not quantified in any way.

We quantified the effect of the anatomical progression and added it to the Results (see below Minor comment #2)

I also found the discussion of audiovisual interactions potentially misleading since there will be correlations in the movie stimuli between auditory and visual features and thus one cannot conclude whether good predictions are driven by acoustic selectivity, visual selectivity or audiovisual selectivity.

We agree with the reviewer and we added these concerns to the Discussion. At the same time we think that training a deep neural network model on the neural responses to a feature film, provides a number of benefits. First, it is a naturalistic stimulus and in real life we often have access to both auditory and visual channels of information. Second, it can easily provide large amounts of data necessary for the model training. Third, one can use the trained model in order to study questions related to multimodal processing and multisensory integration. Specifically, we mention in the Discussion that the trained model weights are available to be used in a new unrelated neural dataset. In this regard, one can apply the pretrained model to data from an experiment addressing the audiovisual interactions with a more constrained experimental dataset.

Given that the primary contribution of this paper, in my opinion, is methodological (not a bad thing in my view), it would be nice for the authors to go into more detail about the various types of model architectures they tried, which types of model architectures worked best (and perhaps why), and any insights they gained along the way. Perhaps the authors could include a summary figure showing the performance of different models along with the spectrotemporal baseline, showing how different architectural choices led to improved performance.

We now report figures comparing the performance accuracy over different architectures of the ANN (**Figure 1b**). We also added a section in Results and Discussion describing the findings: section Architecture of the BO-NN model (pp. 4-5 and p. 14).

I think it might be useful to have a table that shows all of the architectural parameters for each layer of the final model (i.e. kernel size, stride, pooling size and dimension, activation function, etc.). The details should be sufficient such that someone else could easily implement the model. Upon publication, code should be made public on some type of repository, so that someone can download it freely (not upon “reasonable request” as is indicated).

**We added a table with the architectural parameters of the BO-NN model: **Table 1.****

**We rephrased the statement and it now reads: ‘The data supporting the current study have not been deposited in a public repository due to the restrictions on public sharing of the patients’ data but are available from the corresponding author on request. The code containing the BO-NN architecture and the details of training is available at** [**https://github.com/Immiora/bonn_auditory_perception_ecog**](https://github.com/Immiora/bonn_auditory_perception_ecog)**.’**

**In addition, we are currently writing a dataset paper, along with which we intend to release the anonymized neural data used in the present study in the open database (**[**www.openneuro.org**](http://www.openneuro.org)**). We are currently working on the regulatory requirements for doing so but this takes considerable time.**

**More minor comments / points of clarification:**

1. Figure 1a which schematizes the model I think could be clearer. My suggestions/questions: For the “spectra” input display a spectrogram so that it is clear what the input is and flip the axes so that time is on the x axis and frequency is on the y axis. For the “time” input put a waveform so that it is again clear what the input is (perhaps indicate that the filter number is 1 for both the spectra and waveform and the channel number is 1 for the waveform). For the lines/boxes that show the windows of convolution I think it would make sense to make the window size proportional to the filter size (i.e. 7x5 should be bigger than 1 x 1). Also, for the spectra stream, why are there blue cubes and colored planes? And why are there three planes/cubes when the filter number is 1? When the two streams are concatenated the time dimension appears to grow while the channel dimension appears to say fixed, which is the opposite of what occurs. In the last plane, it would be useful to indicate that 396 corresponds to the number of electrodes. How many recurrent layers are there? I would guess one recurrent layer followed by a 1x1 convolution layer to remix the outputs. But from the figure it is unclear. Also, when the authors say the “top layer”, I’m guessing they mean the one right before the final output used to predict the ECoG responses. It would be helpful to clarify this and perhaps make a distinction in the figure between the top layer and the final output.

**We adjusted the diagram of the architecture of the BO-NN model. We added visualizations of sound waveform and sound spectrogram. We flipped the axes so that time domain is on the x-axis. Because the time-domain stream lacked the frequency axis, we thought that it would make more sense to show filter dimension on the y-axis, and for consistence did the same in the spectrogram stream. The frequency dimension in the spectrogram stream is therefore shown along the remaining axis. When the outputs of both streams are concatenated, the filter dimension becomes 400 (from concatenating 300 time and 100 spectrogram stream features), and the time dimensions remains 300.**

**The size of the blocks was adjusted to represent difference in number of filters and amount of time points. We also added a legend for the colors used in the diagram. We indicate that the output dimension of 396 elements corresponds to the number of ECoG electrodes. There is one recurrent layer, and this should now be visible from the color legend.**

**We added clarifications to the figure legend and the main text explaining that top-layer refers to activations of the recurrent layer: “*Activations of the top layer of the model (recurrent layer) were best at predicting the neural responses…”* (p. 6) and “*Next, we sought to understand how our BO-NN features in the top layer (recurrent layer of BO-NN)…”* (p.7).**

2. Figures 4 & 5 are mostly descriptive, with little in the way of quantification, and there isn’t a clear result that one can draw from either. Maybe it would help to combine Figure 6 and Figure 4, and Figure 5 with Figure 7. Figures 5a and 5b don’t seem necessary to me (and plotting the max weight seems like an odd/un-robust statistic, which perhaps explains the noisy-looking pattern). Some quantification of anatomy seems important if the authors want to make anatomical claims. I don’t find the figures particularly compelling.

We appreciate the comment and agree that these figures are more descriptive rather than quantitative. At the same time, we think that in order to offer first insights about the interpretation of this new type of features, it can be particularly beneficial to share the shapes of their time courses and distribution of their cortical profiles. The feature time courses in Figure 4c are discussed in the context of the visible changes in the audio spectrogram, whereas the feature time courses in Figure 5a are described in the context of the neural tuning and their individual weights on the cortex. We believe both are important for feature interpretation and will be interesting for the audience of the PLoS Comp Bio journal.

Regarding the anatomical progression, we carried out a formal analysis that showed evidence of the center of mass in the cortical weights moving more anteriorly in key features #1 and #2 compared to key features #41 and #42 (OLS fit, $F=14.32, p=2\times{10}^{-4}$):

We added the results of the analysis to the Results section (p. 13):

*“Looking at the cortical maps of these features, we could also observe a trend for the electrode locations to move anterior along STG towards IFG. The trend was quantified with an ordinary least squares fit on the key features from Figure 7c and the y-coordinate (posterior-anterior direction) for the center of mass of the cortical weights per feature:* $F(179,1)=14.32, p=2\times{10}^{-4}$ *(see Methods for details).”*

3. For Figure 7, why do the authors switch to using generic features from Praat, instead of the spectrotemporal features. If the authors are worried about delays in the spectrotemporal features, they could simple shift the filters so that they are centered/non-causal. Binarizing the autocorrelation function seems a bit strange to me. Why not just plot the ACF function and put dotted lines at the 50% mark? It would be nice to have a figure that quantifies the relationship between the autocorrelation width and delay, as well as the temporal modulation tuning and delay (a relationship suggested Figure 7c).

We updated the autocorrelation plot by displaying the continuous traces and highlighting the time point when the autocorrelation drops below 50%. In addition, we now report the Spearman correlation between the autocorrelation value and the peak lag for predicting the speech features (PRAAT features) in p.12:

*“Notably, the autocorrelation profiles and the preferred latency were decoupled for many key features (Spearman correlation over all 53 features between the timepoint when the autocorrelation drops below .5 and the latency of tuning to basic speech properties:* $\rho=.2, p=.17$*)”.*

Regarding the PRAAT features, we considered various properties of speech such as sound intensity, pitch, ON/OFF phrasal structure and so on, to be another source of information for interpretation of the BO-NN features. Several of these features also relate to the SMTF information (e.g. pitch and formants). However, in itself they are more interpretable and more relatable to the perceptual properties of speech sounds.

4. It was not clear to me how the RSA analysis was done. Typically one correlates the response pattern across the brain or across a model for pairs of stimuli. Then one correlates this stimulus distance matrix across models/brain regions. Here, they have a time-varying stimulus, so could in principle do this by comparing pairs of time-points, yielding a time-point x time-point matrix which can then be compared across models/regions. It seems like this is not what was done, and I couldn’t figure out exactly what was done and it how it relates to standard RSA.

The RSA analysis was performed over time points as our continuous stimuli did not have a trial structure suitable for a classical RSA. We added more details to the section explaining the RSA analysis in the Methods (p. 26):

*“The RSA was performed separately for the speech and music fragments to account for the possible difference in representation of different types of sound. All sound-specific fragments were concatenated in time and split into 6-second chunks (300 time points at 50 Hz), resulting in 30 speech and 30 music chunks. Then, per chunk, the first order dissimilarity matrices were constructed by computing* $1-\rho$ *(Spearman correlation) across the 6 seconds by correlating feature vectors in each feature set (candidate datasets) and the neural responses in STG electrodes (target dataset) across time points of the chunk. All STG electrodes (199 in total) were used as features of the STG representation. The procedure resulted in 30 matrices (one per 6-second chunk) of size* $300\times300$ *per dataset, each containing dataset-specific pairwise dissimilarities between time points within the chunk. Then, per chunk, the second order dissimilarity matrix was constructed by vectorizing values above the main diagonal (*$44850$ *values) from first order dissimilarity matrices (30 matrices per dataset, target and candidates) and computing* $1-\rho$ *between the datasets. The resulting dissimilarity values (*$1-\rho$*) were compared between the target dataset and each of the candidate dataset in all 30 sound-specific chunks for both speech and music.* ***Essentially, in this approach each time point corresponds to a ‘trial’ and each 6-second chunk corresponds to a ‘subject’ in a standard RSA setting with the difference that the ‘trials’ (time points) are not the same across the ‘subjects’ (6-second chunks).*** *In this setup, the brain data from real subjects with STG electrodes was aggregated together (199 electrodes in total) and treated essentially as coming from the same subject and thus making one (target) dataset. The closest match to the target dataset was determined via a non-parametric two-sided Wilcoxon signed test using a dissimilarity value per each chunk as a single data point.”*

5. In the first movie dataset, used for training, the authors show comparable prediction accuracy for different categories, but in the second movie, performance for music is worse than performance for speech. There is no explanation or discussion of this difference.

We believe that lack of generalization may be explained by differences in HFB cortical responses to music in Movie II due to attention shifts and other top-down processes. Specifically, soundtrack of Movie II had a block design and the subjects could essentially predict the switches between music and speech fragments. Speech fragments contained dialogues relevant for the development of the story whereas music pieces did not.

We added the following paragraph to Results (p. 6):

*“Prediction of music fragments was less accurate and the sound selectivity analysis showed that 83 electrodes in STG and IFG were fitted significantly better during speech compared to music fragments with no electrodes fitted better during music. This difference in prediction accuracy over sound types compared to the results in Movie I was not due to the difference in the music material across the Movies as the acoustic similarity of music across the Movies (*$\bar{s}_{music(I, II)}=89\pm.03$*) was not significantly different from the acoustic similarity of speech (*$\bar{s}_{speech (I, II)}=91\pm.02$*), see Methods for details). Instead the difference was possibly due to the attentional mechanisms arising from the experimental manipulation of the soundtrack in Movie II. Specifically, the sound of Movie II had a predictable structure of interleaved 30-second blocks of speech and music. The speech blocks contained dialogues relevant for the development of the story whereas music blocks did not. This could have triggered attentional shifts between the speech and music blocks, which affected the HFB responses to music. Of interest, the amplitude of the HFB responses to speech in the perisylvian cortex was on average higher compared to music (signed* $\bar{r}^{2}=.77$*, see Methods for details).”*

6. It would be nice if Figure 3b also showed prediction accuracy scores for STG. For this analysis how were the electrodes selected? Hopefully not based upon prediction accuracy of the DNN model as this would be biased?

We added a plot of prediction accuracy in STG to Figure 3a. The details of electrode selection are added to Methods (p. 27): *“Only electrodes with significant cross-validation performance in* ***any*** *of the three models (as determined by non-parametric testing based on shifted data, n = 1,000, see above) were included in the analysis.”*

7. For the spectrotemporal model, I think it would be useful to give the equations for the filters in the text. I know that this is published elsewhere, but it doesn’t take up much space and it’s useful to be sure about exactly what was done.

We expanded on the details of how the STMFs were computed (Methods, p. 25):

*“At the first stage, the auditory spectrogram of the sound is calculated by modeling auditory responses of the cochlea and the midbrain. This process involves a pass of the audio signal through a bank of frequency filters, followed by the transformation into the auditory nerve pattern through a non-linear compression and a membrane-leakage low-pass filter, followed by a pass through a lateral inhibitory network of the cochlea nucleus and signal integration using Gaussian windows of* $8$ *msec mimicking the processing in the midbrain.*

*At the second stage the STMFs are computed from the previously extracted auditory spectrogram reflecting the spectrotemporal analysis postulated to take place in primary auditory cortex. The auditory spectrogram is decomposed using a bank of 2D filters of predefined sizes and orientations, with the two dimensions corresponding to temporal modulations (TMs) and spectral modulations (SMs), respectively. For details of the computational mechanisms and associated mathematical equations we refer the readers to the original paper on STMFs^2^. In the present work we used the STMF implementation provided in the NSL toolbox^2^ and only changed the parameters configuring the STMF filters.”*

The STMF equations and associated derivations would require a considerable amount of space. Since here we used an existing STMF implementation (NSL toolbox), provided by the authors of the STMF model, we opted for referring the readers to the associated paper where they can find the details of the mathematical equations and the relevant derivations.

8. When examining speech/music selectivity it seems more sensible to use a measure like d-prime that incorporates the within vs. between category variance rather than “mean activation”.

We reasoned that the d-prime score is more suitable for discrete responses, such as ‘Yes’ and ‘No’ responses of participants in an experiment. In our case we work with continuous activation values over long time periods. Therefore we decided that parsing the signal into shorter fragments (of 6 seconds) and calculating the average difference in activation between speech and music parts of the signal would be more suitable.

9. Preference constant is not defined.

We adjusted our description of the preference parameter (p. 27):

*“The optimal number of clusters was identified in a data-driven way by varying the parameter of* ***preference*** *that represents the initial suitability of each data point to be a cluster center. By default is AP clustering preference value is set to the median of the similarity matrix (or affinity matrix). Here, instead of using the default we varied the value of preference until the estimate of the resulting number of clusters fell in the knee of the curve, representing the optimal balance between maximum compression and optimal cluster assignment accuracy. “*

10. I assume that nested cross-validation means that there was cross-validation within the training set to pick the regularization parameters. It would be nice to spell this out and give the details (i.e. the number of folds for the inner and outer loop).

We made the details of training more explicit (p. 22):

*“The optimal value of the regularization parameter* $\lambda$ *(equation 4) was determined through a nested cross-validation (k = 10) […] The model performance was cross-validated across six 30-second test folds (*$k=6$*).”*

11. It would be nice to give statistics about the amount of speech, music and other categories present in the training movie.

We added a summary of sound diversity of Movie I in **Figure 2a**.

12. The parametric t-stat computation shown in Figure 2 seems inappropriate since n presumably reflects the number of timepoints (not specified), but adjacent timepoints are presumably not independent.

We provided parametric results when testing significance of model performance in held-out test data of Movie I (new Figure 2b). This was done because of the high computational demands associating with non-parametric testing based on retraining the BO-NN model on permuted or shifted data a sufficiently large number of times. Training of one instance of the model (ten cross-validation folds trained simultaneously) required approximately 30 hours on our CPU system (training on a GPU was faster but training ten folds had to be consecutive and resulted in a similar amount of time). We understand that parametric testing leads to an unreasonably low threshold on the performance accuracy and for this reason set the $p$-value to a very small number: $p<1\times{10}^{-20}$ at least. Importantly, all model comparisons yielded the same results regardless of the chosen $p$-value: $1\times{10}^{-20}$, $1\times{10}^{-50}$, $1\times{10}^{-80}$ or $1\times{10}^{-100}.$

When assessing the generalization performance of the model on data of Movie II we used non-parametric testing. For the regression trained on input data (STMFs, random-NN or top BO-NN features) to predict HFB ECoG responses we permuted sound blocks in input data and shifted them by a randomly chosen number of time points (minimal shift of 20 seconds to account for autocorrelation) and then trained the regression. Repeating the process 1,000 times yielded a baseline distribution of model performance. We used the 99.999^th^ percentile of this baseline distribution, which corresponds to a $p$-value of $.001$, to threshold the performance accuracy achieved with the regression on actual, non-shifted data.

13. The color-map in Figure 1 is hard to compare with the balls in the brain. Perhaps the authors could replace the color-bar with balls that have corresponding correlation values for easier comparison.

We think that it is quite a standard way to represent ECoG results and would like to keep this format. However, we want to thank the reviewer for this comment as it drew our attention to the fact that very dark red on the colormap (low but significant correlations) looked very similar to black (electrodes with a non-significant fit, plotted for coverage reference). We replaced the colormap by removing very dark red from it and reduced the marker size for the non-significant electrodes. We hope that this makes the plot more readable.

14. Were the electrode responses normalized or z-scored for any of the analyses?

Both sound and ECoG data were normalized prior to the BO-NN training and also for the analyses on the generalization of the model performance with Movie II. We added this information to the Methods (pp.21, 23):

*“The input (audio) and output (HFB ECoG) data were first preprocessed using a standard scaler from scikit-learn (Pedregosa et al., 2011) that z-scored the data per channel (per frequency bin of spectrogram and per ECoG electrode in brain data).”*

*“Both sound features extracted at each layer of the BO-NN model and the HFB responses to Movie II were z-scored prior to the ridge linear regression fit.”*

15. There is no description or reference to the kernel density estimator used and how it was applied (i.e. Figure 6b). Also in Figure 6b, does each line correspond to the correlation value for the spectrotemporal feature with the highest correlation for a single “key feature”? If so, it would be good to clarify this in the legend.

We updated the plot in Figure 7b and separated the kernel density estimation plots from the correlation plots to make the graphs more readable. Per BO-NN feature, we find an STMF with the most similar time course. Each STMF is a combination of values along three dimensions: spectral modulations (SMs), temporal modulations (TMs) and frequency. Therefore, per BO-NN feature we plot its correlation (a dotted line) for the value of each STMF dimension separately. The procedure is done for speech and music separately.

Above the correlation plots we now also show the Gaussian kernel density plots and the rug plots for the STMF values that individual key BO-NN features are ‘selectively tuned to’. Effectively, instead of associating each BO-NN feature with a combination along all three STMF dimensions we estimate which of the three dimensions the given BO-NN feature is most selective to (see the legend and the description in Methods, p.29). It turns out that there is less selective tuning to the spectral modulations in speech than the temporal modulations, possibly because the variation along the dimension of the temporal modulations is likely linked to the language units of varying lengths (such as phonemes, syllables, words and phrases).

The density plots were computed based on a Gaussian kernel density fit with default density estimation settings provided by the *statsmodels* ([www.statsmodels.org](https://www.statsmodels.org/)) Python library. We added this information to the Methods and the figure legend.

16. Why did the authors use the beta weights to cluster the features as opposed to the similarity of the response timecourses? Perhaps there could be features with very different response selectivity but similar beta weights such that averaging these features into one cluster would be inappropriate?

We opted for clustering of the weights so that we could use the anatomic consistency as an additional source of information for the feature interpretation. We used this approach previously in the paper on STMF encoding in the perisylvian cortex (Berezutskaya et al., 2017) so we considered it a suitable method for interpretation of cortical tuning to features of the BO-NN model.

Besides, the affinity propagation clustering provides cluster centers by selecting the most representative data point for each cluster without averaging of time courses over features that belong to the same cluster.
