# Supplementary material for: Brain-optimized extraction of complex sound features that drive continuous auditory perception
Source: PLoS Comput Biol. 2020 Jul 2;16(7):e1007992. doi: 10.1371/journal.pcbi.1007992 (PMC7363106; doi:10.1371/journal.pcbi.1007992)
Supplement: Attachment — Submitted filename: response_to_reviewers_second_round.docx [file pcbi.1007992.s003.docx]

**Reviewer #1**

In this revision of “Brain-optimized extraction of complex sound features that drive continuous auditory perception”, Berezutskaya et al. use a “brain optimized neural network” (BO-NN) to discover features driving neural activity recorded from ECoG during a movie watching task, and then use those features to predict neural data recorded from different participants on a separate natural listening task. Their main findings are that they can use features from a recurrent convolutional neural network to extract feature representations that can be used to predict neural data on a separate cohort of patients, and that there are some separable effects for speech and music in different anatomical areas. My previous review addressed a lack of interpretation of the “key features” from their models, and a need for direct comparisons between these and the spectrotemporal models. I believe this has been addressed nicely in the new revision of the manuscript. They have also included more details on the stimuli, including amount of speech, music, and other sounds (also requested by Reviewer #3). I agree with Reviewer #2 somewhat about point #3, which is that “it is interesting to show what is added by the network that wasn’t in the theory-driven features”. Still, I think the authors respond well by noting that interpretation of neural network models is often difficult, and I think they’ve done a good job at describing why the BO-NN might be better. Even if this paper doesn’t tell us exactly what is being represented by the NN, that is similar to many artificial neural network papers and is not unique to these authors, so I wouldn’t say the burden of proof is on them to show this. Still, I think it’s up to the reader (and future experiments) to determine what exactly is missing from our theories about what these areas “do” or “represent”!

I only have a few minor comments, which would not preclude acceptance but could be fixed in the final version:

New Figure 2:

• There is a small typo in the title, “Distritbution” should be “Distribution”.

We fixed the typo and uploaded the corrected figure.

• The semicircular plots are fine and aesthetically pretty, but I think the result would be clearer as a standard bar plot.

Given the space limitations and the amount of information we wanted to display, we considered the half-pie charts more appropriate.

**Reviewer #3**

The authors have made a number of efforts to clarify the methods of the paper. I particularly appreciated the dramatic improvements to Figure 1, which is both much clearer and provides some useful insights as to which aspects of the model improved performance.

I continue to think this paper represents a useful methodological advance, and is worth of being published as a methods paper. However, the scientific claims are not supported by the data. The key claims from the abstract are:

*“Several features encoded speech-specific acoustic properties with some features exhibiting shorter latency profiles (associated with responses in posterior perisylvian cortex) and others exhibiting longer latency profiles (associated with responses in anterior perisylvian cortex). Our results support and extend the current view on speech perception by demonstrating the presence of temporal hierarchies in perisylvian cortex and involvement of cortical sites outside of this region during audiovisual speech perception.”*

1. What is the evidence for speech-specificity? The fact that a few electrodes on average respond preferentially to speech over music (Fig 5b)? What representation wouldn’t show this? I suppose there is the fact that the model better predicts speech in Movie II, but it’s unclear what to make of this, especially given that the same is not true in Movie I.

We softened the claim in the abstract:

*Specifically, several features encoded* ***speech-related*** *acoustic properties with some features exhibiting shorter latency profiles (associated with responses in posterior perisylvian cortex) and others exhibiting longer latency profiles (associated with responses in anterior perisylvian cortex).*

Regarding the differences between Movie I and II, we address the issue in the Results and suggest that the observed difference is likely due to the attentional changes. Even though an in-depth analysis is outside of the scope of this work, we mention briefly in the Discussion that attention has a great effect on the neural responses in low-level perceptual cortices and modifications to ANN approaches should be considered, so that they attempt to incorporate high-level processes in modeling the brain activity based on perceptual input.

2. With regards to the posterior-to-anterior hierarchy, the only quantitative data plotted is in the figure below, which is from the response and not even included in the paper. The figure shows a trend line for five hand-picked features. The trend line looks like it accounts for a tiny fraction of the variance, and seems to be driven by maybe the first or second feature.

Instead of hand picking the features we adopted a more structured approach. We describe the approach and the results on pp.14-15:

*Finally, we sought to combine together the results we had obtained from the previous analyses aimed at interpreting the BO-NN features. We constructed a matrix that contained per key BO-NN feature the information about its correlation with the SMTFs, its prediction accuracy from the linear fit using the Praat features and the magnitude of beta-weights from the linear fit from top BO-NN features to the ECoG responses. We also added information regarding the location of the electrodes with high beta-weights, such as the percentage of the electrodes in the perisylvian cortices and a ratio of the electrodes in the perisylvian cortex to the electrodes in other brain areas. We focused on the perisylvian cortex because the prediction accuracy in these regions was best for the BO-NN model compared to the control models (****Figure 4****) and the majority of the electrodes that were associated with the BO-NN features were located there (****Figure 6b****).*

*Then, we used multidimensional scaling (MDS) to visualize the feature space according to the results of our previous analyses and the relationship to the activity in the perisylvian regions (****Figure 8c****). We observed that each MDS dimension was highly interpretable: MDS dimension 1 was most related to the correlation with SMTFs, MDS dimension 2 was most related to the results of the fit on Praat features and MDS dimension 3 was most related to the anatomical constraints and the relation to the brain activity in the perisylvian regions. As a result of this projection, we also observed that a subset of key BO-NN features exhibited high values along all three MDS dimensions: #41, #42, #17, #16, #49, #36, #1, #22 and #2 (****Figure 8c****).*

*Previously we observed a trend for an increased latency of response for these features: from -50 – 0 ms prior to speech onset (key feature #41) towards 250 ms after speech onset (key features #1, #22, #2,* ***Figure 8c****). Looking at the cortical maps of these features, we could also observe a trend for the electrode locations to move anteriorly along STG towards IFG. Therefore, next we tested the theory about the posterior-anterior gradient of temporal information encoding along the perisylvian cortex (Berezutskaya et al., 2017; Binder et al., 2000; Hullett et al., 2016). We sorted the selected BO-NN features (highlighted in* ***Figure 8c****) according to their temporal response profiles (****Figure 8b****) and used them to fit the y-coordinate (posterior-anterior direction) for the center of mass of the cortical weights within the perisylvian regions. This fit was significant:* $F(313,1)=28.02, p=2\times{10}^{-7}$ *(ordinary least squares fit,* ***Figure 8d****), supporting the idea of the posterior-anterior anatomical progression for the BO-NN features that were most correlated to acoustic STMF characteristics, were well fitted by sound intensity, pitch and speechON features, and were localized in the perisylvian cortical network. Importantly, this posterior-anterior gradient was associated with tuning to increasingly slow TMs (****Figure 8d****), providing data-driven evidence for the theory of the temporal gradients along the perisylvian regions involved in speech perception (****Figure 8e****).*

We also updated the **Figure 8** accordingly by adding panels **c, d** and **e**:

The legend of **Figure 8** was updated accordingly (p. 54):

*[…]*

***(c)*** *The results of the MDS for the key BO-NN features. Various information was used for the MDS including the results of the previous analyses (correlation to STMFs, linear fit using Praat features), the magnitude of the associated cortical weights and the anatomical constraints (association with cortical weights specifically from the perisylvian regions). Each circle represents one key BO-NN feature, the labels correspond to the indices of the key features. The cluster of features with the highest values along all MDS dimensions is highlighted in red. Per MDS dimension we also show its maximal correlation with the specific type of information used in the analysis (e.g. correlation with STMFs).* ***(d)*** *Left plot shows the results of the ordinary least squares fit of the sorted key features (highlighted features in* ***c****) to the y-coordinate (anterior-posterior direction) of the center of mass for the ECoG electrodes associated with each feature (based on the beta cortical weights). The boxes show the 25^th^ and 75^th^ percentiles of the electrode y-coordinates per feature, caps show 5^th^ and 95^th^ percentiles. A solid line in the middle shows the median. The line across the boxplots represents the result of the ordinary least squares fit. The slope and intercept of the fit are also reported on the top of the plot. Right plot show gradual decrease in the TM tuning for the features highlighted in* ***c*** *as well as the gradual increase in the temporal response profile (i.e. optimal shifts for the prediction of the key BO-NN features using Praat features shown in* ***a****).* ***(e)*** *A tentative timeline of neural processing of audiovisual speech in -50 to 250 ms around the sound onset. The plots contain cortical weight maps for selected key features (highlighted in* ***c****) together with their example time course during a speech fragment. Only the electrodes from the perisylvian cortices are shown, the weights of the electrodes in the right hemisphere are mapped onto the analogous location in the left hemisphere.*

3. Finally, the “audiovisual speech” effect could be driven entirely by correlations in the stimulus set between visual and auditory features (i.e. the neural responses themselves might be entirely tuned to one modality alone). The fact that the effect cannot be explained by differences in luminance is uncompelling as the visual cortex responds to an enormous range of visual features beyond luminance.

We softened our claims regarding the audio-visual interactions and stated that more work focused specifically on this topic is required (p.18 of Discussion):

*We believe that these distinctions are unlikely to represent low-level visual confounds, but rather reflect high-level interactions […] More work focusing on the audio-visual interactions as well as more comprehensive control for the low-level visual confounds will be needed to add to this discussion.*

We are also currently working on another manuscript that uses these data, where we show that high-level semantic features trigger the responses of many cortical areas including lateral occipitotemporal complex, frontal and parietal regions. In addition, in that manuscript we discuss that both perceptual modalities (auditory and visual inputs) exhibit interactions with the higher-level content, which we find not to be surprising for a naturalistic stimulus. There we also perform a more rigorous comparison with several control low-level visual feature sets, including pixels values, Gabor features and various layers of a deep neural network for object recognition. Notably, it was the involvement of the occipitotemporal region in the present results that stimulated our interest in further exploration on these data with respect to the encoding of the visual and high-level semantic properties.

4. The paper would be much stronger in my opinion if it was purely methodological. The scientific claims are not supported by the data, and water down an otherwise useful methodological advance.

We agree that our manuscript has a significant methodological weight to it. However, we argue that efforts to interpret results in neuroscientific terms constitute an important step for adoption of deep learning approaches to the highly complex nature of human cognition. We believe we promote this cause by presenting efforts towards interpreting patterns emerging from the analyses, and showing that results are in line with results presented in top-down, theory-driven approaches

--

5. I have one other substantial methodological comment. When describing the STMF baseline, the authors note that they considered a wide range of parameters for frequency, temporal and spectral modulation. But the authors do not say anything about including temporally delayed copies of the response, which is obviously important when trying to predict a time- varying signal. Can the authors comment on this, and if necessary run a version of the analysis that includes temporal delays?

We want to thank the reviewer for this suggestion. We ran the analysis allowing the STMF model to use features at multiple delays. We then compared the prediction accuracy of the fit using 300 top-layer BO-NN features (no delays) against the fit using 1768 STMFs included at different temporal delays (13 values along the spectral modulation dimension, 17 values along the temporal modulation dimension and eight values along the frequency dimension as explained in Methods p.29).

Methods (p. 30):

*In addition, we trained another ridge linear regression model using STMFs at multiple temporal delays with respect to the audio onset to compare it against the linear model using BO-NN features that did not incorporate any temporal delays. At each time point within 500 ms from the audio onset a vector of 1768 STMFs was added to the model following the setup from previous studies (Berezutskaya et al., 2017; Pasley et al., 2012). At 50 Hz this resulted in 44,200 features to predict ECoG responses per one timepoint. The linear models using both feature sets (STMFs at various temporal delays and BO-NN at no temporal delays) included a regularization parameter to improve the fit and generalization. The linear model training and evaluation were performed in the same way as described previously, by employing a nested cross-validation to estimate the regularization parameter and computing prediction accuracy by cross-validating per-block performance. The cross-validated performance was compared between the two models using two-sided Wilcoxon signed test per ROI as described previously.*

Results (p. 10):

*Importantly, we observed that the fit using only 300 BO-NN features was superior to the STMFs even when aggregating STMFs over multiple temporal delays within 500 ms from the audio onset (see Methods for more details). The improvement was significant for STG* $(Z_{BONN-STMF\_DELAYS}=3.93, p=8\times{10}^{-5}$*, +7 sign. els.) and MTG* $(Z_{BONN-STMF\_DELAYS}=2.15, p=.03$*, +2 sign. els.).*

At the same time, we think that one of the strengths of the DNN approach to extract optimal features is in its capacity to learn the latencies of the neural response as well. This method relieves the researcher from finding the optimal lag per each channel of the brain data, as well as deciding on what range of lags is reasonable to consider. In that regard, we believe it is fair that both feature sets are fed into the linear regression model timepoint- by-timepoint at no audio-ECoG delay. It is the advantage of the DNN that it incorporates the temporal response function learnt directly from the data. And it is even more convincing that these temporal response functions were learnt on a separate set of audio-ECoG data, yet appear to generalize very well.

We added a statement about it to Discussion (p. 17):

*Notably, the superior prediction accuracy achieved by the BO-NN model could not be reproduced simply by aggregating STMFs over various temporal delays. At the same time, we would argue that the ANN’s inherent ability to learn the temporal function of the neural response directly from the data is in itself superior to the methods that require aggregation of features at multiple lags.*

6. I have a similar worry with the RSA analysis (which the authors have done a nice job of clarifying). Since the model and brain data are being compared timepoint-by-timepoint, trivial differences in delays could explain the better performance of the DNN model. Or perhaps the STMF just doesn’t have the right proportion of each type of spectrotemporal modulation filter? The authors could try and address this by performing RSA after a linear transformation to align the model with the data. But I honestly don’t see any benefit of the RSA analysis over simply predicting the response.

As we mentioned above, it requires extra work to determine the optimal lag for each of the ECoG channels when using STMFs. In addition, we are not sure how to incorporate the shifts for STMFs that would surely vary across ECoG STG electrodes (as we saw in our previous publication: Berezutskaya et al. 2017) in the current implementation of the RSA. Besides, the results of the analysis following the suggestion in the previous comment (where we compared the prediction accuracy of STMFs accumulated over a 500 ms window and the top-layer BO-NN features with no additional temporal delays) once again indicate that our DNN features provide a better representation of the brain responses compared to STMFs.

Minor comments:

1. In Figure 7b, the kernel density plots don’t seem to reflect the correlation plots below. For example, the spectral modulation correlations appear broadly distributed in the lower plot, but peaked in the kernel density plot.

We split the **Figure 7b** into sections **b** and **c**, as the probability distributions (former top plots) and dotted lines (former bottom plots) show different types of information. We also added more explanation to the figure legend (pp. 52-53):

*This distributions of dimension selectivity for all key BO-NN features with significant correlations to STMFs are shown in the rug plots and the probability density plots above the correlation profiles per STMF dimension and sound type. Thus, the distribution plots show information that is different from the information shown on the correlation plots (dotted lines). In the example of the key BO-NN feature #2 the dimension selectivity is shown as a solid line in the correlation plot. It is also displayed in the rug plots and the probability density plots above the correlation plot. Overall, the plots show that BO-NN features were more selective to the dimensions of the temporal modulations and frequency (as there was more variance in correlation for the different STMF values along these dimensions), whereas the dimension of the spectral modulations while showing high BO-NN-STMF correlations was associated with less variance in correlation along the different STMF values and therefore less preference in BO-NN features to its specific values.*

2. Why go with the dual stream model as the main model, given that it is more complex, and doesn’t seem to predict responses better? The authors say “The reason for that was that we aimed to maximize the generalization capacity of the ANN and considered that both types of input may carry information that could aid generalization.” But they do not provide any evidence that the more complex model aids in generalization.

The actual analysis comparing the fit using different types of audio input did not show any significant difference between the models. Based on some previous literature (Güçlü et al., 2016; Lim et al., 2018), the dual-stream model seemed to be the best candidate. We would leave it up to the reader to decide on the implications of the presented model comparisons.

In principle, the information in the frequency and time domains of the audio is equivalent and given the sufficient amount of data, the optimal transformations needed for the best fit of the brain data can be performed on a single audio stream. Because the data of the Movie II might have been different enough (for example, we saw the possible effects of the attentional shifts there), we aimed to keep as much information about the input data as possible to make it easier for the model to generalize. Therefore, we went with the dual-stream model.

3. The authors’ measure of speech and music selectivity is still suboptimal, since it doesn’t obviously measure whether the responses are consistently higher or larger for speech or music. I suggested using d-prime, but the authors responded saying: “We reasoned that the d-prime score is more suitable for discrete responses.” What I meant was to simply measure the difference in the means divided by standard deviation of the response within each category, which is a very standard way of measuring the amount of overlap between two continuous distributions.

We calculated the d-prime statistics and added the results to the manuscript:

Results (p. 11):

*Statistical testing showed that some of the key BO-NN features were specific to speech (e.g. key features #1, #2 and #36), some were sensitive to both speech and music (e.g. key feature #42) and some showed enhanced response to music (key features #40, #48 and #15), as assessed using a d-prime (*$d'$*) statistic for signal separability (Macmillan and Creelman, 2004) (****Figure 5b,*** *see Methods for more details).*

Methods (pp. 31-32):

*In order to test feature specificity to speech or music, we calculate a d-prime (*$d'$*) statistic (Macmillan and Creelman, 2004) per feature. The statistic assesses the degree of separability of two signal distributions and in our case, the separability of speech and music activations per feature:*

$$d^{'}=\frac{\mu_{s}-\mu_{m}}{\sqrt{\frac{1}{2}(\sigma_{s}^{2}+\sigma_{m}^{2})}}$$

*(5)*

*where* $\mu_{s}$ *and* $\sigma_{s}$ *are respectively the mean and the standard deviation of the feature’s activation during speech fragments, and* $\mu_{m}$ *and* $\sigma_{m}$ *are respectively the mean and the standard deviation of the feature’s activation during music fragments.*

*We then performed the significance testing by constructing surrogate distributions of* $d'$ *values per feature computed on permuted activation time courses. For that, speech and music blocks were permuted, and the activation time courses were recomputed per feature. Then, the* $d'$ *statistic was calculated for each permuted time course. We used 10,000 permutations per feature. The actual* $d'$ *values computed on original, non-permuted time courses, were considered significant if they fell in either above the 99^th^ (specificity to speech) or below the 1^st^ (specificity to music) percentile of the surrogate distributions. These values are highlighted in* ***Figure 5b.***

Figure 5b:

Legend for Figure 5b (p. 50):

*Top plot shows key BO-NN features with maximal average activation across speech or music fragments. Bottom plot show music-speech specificity values per feature as assessed with* $d'$ *statistics (signal separability index). Boxplots show surrogate distributions used for significance testing (obtained by permuting speech and music blocks and recalculating* $d'$ *values per feature 10,000 times). The boxes show the 25^th^ and 75^th^ percentiles of the surrogate* $d'$ *values computed on permuted feature activation time courses, caps show 1^th^ and 99^th^ percentiles. A solid line in the middle shows the median. The actual* $d'$ *statistics are shown as circle markers per feature. The markers are filled if the actual*$d'$ *statistics fall above the 99^th^ (red markers, speech specificity) or below the 1^st^ (blue markers, music specificity) percentile of the surrogate* $d'$ *distributions.*

4. There are small grammatical and text errors throughout. I have not noted them all because this would have been time consuming, but hopefully the paper could be carefully proofread before publication.

We thank the reviewer for pointing this out. The final version of the manuscript was proofread by two native English speakers.

**REFERENCES**

Berezutskaya, J., Freudenburg, Z.V., Güçlü, U., van Gerven, M.A., Ramsey, N.F., 2017. Neural tuning to low-level features of speech throughout the perisylvian cortex. J. Neurosci. 37, 7906–7920.

Binder, J.R., Frost, J.A., Hammeke, T.A., Bellgowan, P.S.F., Springer, J.A., Kaufman, J.N., Possing, E.T., 2000. Human temporal lobe activation by speech and nonspeech sounds. Cereb. Cortex 10, 512–528. https://doi.org/10.1093/cercor/10.5.512

Güçlü, U., Thielen, J., Hanke, M., van Gerven, M.A.J., 2016. Brains on beats, in: Lee, D.D., Sugiyama, M., Luxburg, U.V., Guyon, I., Garnett, R. (Eds.), Advances in Neural Information Processing Systems 29. Curran Associates, Inc., pp. 2101–2109.

Hullett, P.W., Hamilton, L.S., Mesgarani, N., Schreiner, C.E., Chang, E.F., 2016. Human superior temporal gyrus organization of spectrotemporal modulation tuning derived from speech stimuli. J. Neurosci. 36, 2014–2026. https://doi.org/10.1523/JNEUROSCI.1779-15.2016

Lim, T.Y., Yeh, R.A., Xu, Y., Do, M.N., Hasegawa-Johnson, M., 2018. Time-frequency networks for audio super-resolution, in: 2018 IEEE International Conference on Acoustics, Speech and Signal Processing (ICASSP). IEEE, pp. 646–650.

Macmillan, N.A., Creelman, C.D., 2004. Detection theory: A user’s guide. Psychology press.

Pasley, B.N., David, S.V., Mesgarani, N., Flinker, A., Shamma, S.A., Crone, N.E., Knight, R.T., Chang, E.F., 2012. Reconstructing speech from human auditory cortex. PLoS Biol. 10, e1001251. https://doi.org/10.1371/journal.pbio.1001251
